# Internationalist blood: Karel Holubec and the diffusion of Duran Jordà’s method of blood transfusion to Czechoslovakia, 1930s–50s

**DOI:** 10.1017/mdh.2024.27

**Published:** 2025-01

**Authors:** Carles Brasó Broggi, Hana Bortlová-Vondráková

**Affiliations:** 1 Department of Arts and Humanities, Universitat Oberta de Catalunya, Barcelona, Spain; 2 Institute of Contemporary History of the Czech Academy of Sciences, Prague, Czech Republic

**Keywords:** Spanish Civil War, International Brigades, Czechoslovakia, Diffusion of medical innovations, Internationalist medicine, Blood transfusions

## Abstract

In the first months of the Spanish Civil War, the Spanish doctor Frederic Duran Jordà developed a new method of blood transfusion which overcame the era of direct arm-to-arm transfusions. While Duran was experimenting in Barcelona and the Aragon front, hundreds of foreign doctors came to Spain with the help of internationalist associations and offered their services to the Republican government. The Czechoslovak Dr Karel Holubec entered Spain in May 1937 and practiced in a mobile hospital funded by the Czechoslovak Committee to Aid Democratic Spain, receiving blood from Duran’s laboratory. This article aims to study how Duran and Holubec transferred the method of blood transfusion to Czechoslovakia through interpersonal contact, conferences, and performances. This paper argues that while individual actors played a crucial role in the diffusion of medical practices, this circulation was determined by a unique historical and socio-political framework. The Spanish Civil War, the International Brigades, and the invasion of Czechoslovakia by Nazi Germany were not only the historical context of medical innovation but an integral part of it.

## Introduction

One of the most relevant medical innovations produced in the Spanish Civil War (1936–39) was the technique of blood transfusion developed in Barcelona by Frederic Duran Jordà (1905–57).^1^ This article analyses how this method was exported to Czechoslovakia thanks to Duran’s travels in 1938 and the disseminating role of Czech doctor Karel Holubec (1906–77). Both Duran Jordà and Holubec were part of larger social movements that called for international solidarity between the countries affected by what they perceived as a common threat of global fascism. The blood transfusion method of Duran Jordà involved the massive participation of blood donors who, in the heated atmosphere of the Civil War, responded to appeals broadcast over the radio. This article claims that Duran’s innovation and its diffusion to Czechoslovakia cannot be detached from this particular context. The historical circumstances of this transfer, as it will be argued, determined both the success of this method and its further oblivion from the medical records.

In March 1937, when the blood donation campaigns in Spain were at their peak, the American doctor Bernard Fantus (1874–1940) introduced another innovative blood transfusion system at the Cook Hospital in Chicago. It was a different context. This was the first blood transfusion institution to be called a ‘blood bank’: the system incorporated banking methods with a system of paid donors where blood would be stored as deposits or taken on credit.^2^ Instead, Duran Jordà relied on mass voluntary donations and internationalist aid. He received ambulances as a gift from the United Kingdom (UK)-based Spanish Medical Aid Committee (SMAC) and, when he travelled to Prague, a campaign to donate Czechoslovak blood to Spain was in full swing. The ideological and political terrain thus shaped the ways in which Duran Jordà and Fantus innovated in the field of blood transfusion. This article focuses on the former and frames the transfer of Duran’s method with internationalism, wartime antifascism, and socialism.

While the biographies of doctors who participated in the International Brigades have attracted academic attention, there is also a rich bibliography concerning the Spanish republican doctors who migrated to other countries carrying their war experiences and know-how.^3^ While this article adopts the circulation of two persons as a methodology of ‘local storytelling’ that, according to Zemon Davis, allows for a decentralized global approach, we are not so much interested in individual agencies and biographies but in the less explored terrain of how the geopolitical environment of two side-by-side conflicts – the Spanish Civil War and the occupation of Nazi Germany of Czechoslovakia – shaped the process of innovation and transfer.^4^

Duran Jordà was born in Barcelona and studied medicine in the Hospital Clínic, a pioneering medical centre in Spain. He graduated in 1928 and worked as a lab analyst attached to the chair of surgical pathology and as a blood transfusion expert at the Maternity Hospital of Barcelona.^5^ After the coup of Francisco Franco in July 1936, he organised a pioneering blood transfusion centre, supported by the Socialist political parties, with the mission to extend blood transfusions to Barcelona and the Aragon front.^6^ In October, Duran Jordà led the Frontline Blood Transfusion Service (Servei de Transfusió de Sang al Front) that was created by the War Medicine Bureau of the Catalan Government (Consell de Sanitat de Guerra) and, in July 1937, the Transfusion Service of the Spanish Republican Army (Servicios de Transfusión de Sangre).^7^ Previous research has analysed how Duran Jordà improved the technique of blood extraction, preservation, and transfusion, with a pre-packed device, enabling the extraction, preservation, and transfusion of unprecedented amounts of blood with greater facility and efficiency.^8^

Holubec was born in Pržno, a small village on the Moravian–Silesian–Polish border, into the Czech evangelical family of a rural teacher. Karel’s father died when he was three years old and the family lived in difficult material conditions. This experience shaped his later ideological attitudes, including his socialist and antifascist views, and his medical career. Thanks to a scholarship for gifted poor students, he was able to graduate from high school and pursue his studies in the capital. He was educated as a surgeon and graduated from Charles University in Prague two years after Duran, in 1930. He directed the Komenský Hospital which entered Spain in May 1937.^9^ This mobile hospital was a donation of the Czechoslovak Committee to Aid Spanish Democracy (Výbor pro pomoc demokratickému Španělsku, VPDŠ). It was a part of a larger international medical aid movement that rose in support of the Spanish Republican government against Franco’s coup and against his Italian and German allies. Most of these international teams, like the Komenský Hospital, joined the sanitary services of the International Brigades: the International Sanitary Service (Servicio Sanitario Internacional). This institution became a disseminator of medical practices, bringing together hundreds of doctors of more than thirty nationalities.^10^

This case study aims to analyse the international diffusion of medical innovations through semi-peripheral channels. It is based on unpublished archival material complemented by contemporary publications in Catalan, Spanish, Czech, French and English. The existing literature on the diffusion of medical innovations tends to focus, on one hand, on the most developed countries and scientific production centres and, on the other hand, on the colonial and postcolonial worlds, leaving aside the relations and diffusions on the semi-peripheries such as Spain and Czechoslovakia.^11^ Between 1937 and 1938, Duran Jordà’s method was published in Catalan, Spanish, French, and English journals but it was not until 1939, after he had migrated to the UK, that his method became well-known to the international scientific audience thanks to an article published in *The Lancet.* ^12^ The transfer of Duran Jordà’s method to the UK has thus been well studied.^13^ However, before that, in 1938, Duran’s method was exported to Czechoslovakia through interpersonal contact, conferences, and demonstrations. This transfer had a massive impact and further influenced Czechoslovak doctors. This article aims to move beyond technological determinism to engage with social and political contingencies that shaped the circulation of this method. Both Duran and Holubec were involved in a highly mobilised political movement that sought to defend both countries from the threats of Spanish Francoism and Nazi Germany. Whereas this movement was unsuccessful, as both countries succumbed to these threats, Duran Jordà’s method was effectively transferred to Czechoslovakia. However, as the post-war evolution of Czechoslovak science gravitated towards Sovietisation, this successful case of knowledge transfer was sidelined from both the international streams of scientific records and the national narrative of Czechoslovak socialist medicine history.

## Duran Jordà and the Komenský Hospital in the Spanish Civil War

Blood transfusions existed since the seventeenth century but they were a tool of last resort and were only used sporadically due to the high risk involved.^14^ In the first decades of the twentieth century, the advancement in the classification of blood groups, and the innovations in anti-coagulants and medical instruments (syringes, cannulas, glass storages), allowed blood transfusions to spread from university surgical clinics and experimental labs to smaller hospitals.^15^ In the mid-1930s, the Soviet doctor Sergei Yudin (transliterated also as Yudine/Judin/Judine) extracted blood from corpses, mixed it with anti-clotting agents, preserved it in refrigeration stores, and transported it with vehicles. He was the first doctor to experiment extensively with preserved blood, documenting a thousand transfusions in 1937.^16^ Furthermore, the Russian surgeon gave a conference at the first institute of blood transfusion in Barcelona created by Dr Antoni Trias Pujol. Duran Jordà, at that time a young doctor in the digestology department, attended the speech and experimented with the extraction of cadaveric blood.^17^ This method was discussed at the First and Second Congress of the International Society of Blood Transfusion celebrated in Rome (1935) and Paris (October 1937).^18^ However, both events gave priority to direct blood transfusions and ignored the importance of preserved blood as late as October 1937; at that time, the innovations of Duran Jordà were only available in a Catalan article.^19^

In the 1930s, Duran Jordà became involved with political movements that mixed socialism with Catalan nationalism. He joined a political party called Unió Socialista de Catalunya (Socialist Union of Catalonia), in which important Catalan socialist leaders like Joan Comorera and Rafael Campalans were members.^20^ Duran Jordà was interested in state-building as a way to implement a socialist health system, especially to protect workers who were unable to afford private medical care.^21^ He followed the studies of the new countries that emerged after the First World War, especially the Soviet Union and Czechoslovakia. According to his friend, the doctor Josep Trueta, Duran Jordà learned from a few studies of preserved blood before the clash of the Spanish Civil War: one was Yudin and the other were publications from Czechoslovakia.^22^ Trueta may have provided this information himself and travelled to Prague in 1932 with a delegation of Catalan surgeons led by Dr Corachán to meet Dr Arnold Jirásek (1887–1960). This delegation was moved by the modernization process of the young state of Czechoslovakia.^23^

**Figure 1.**
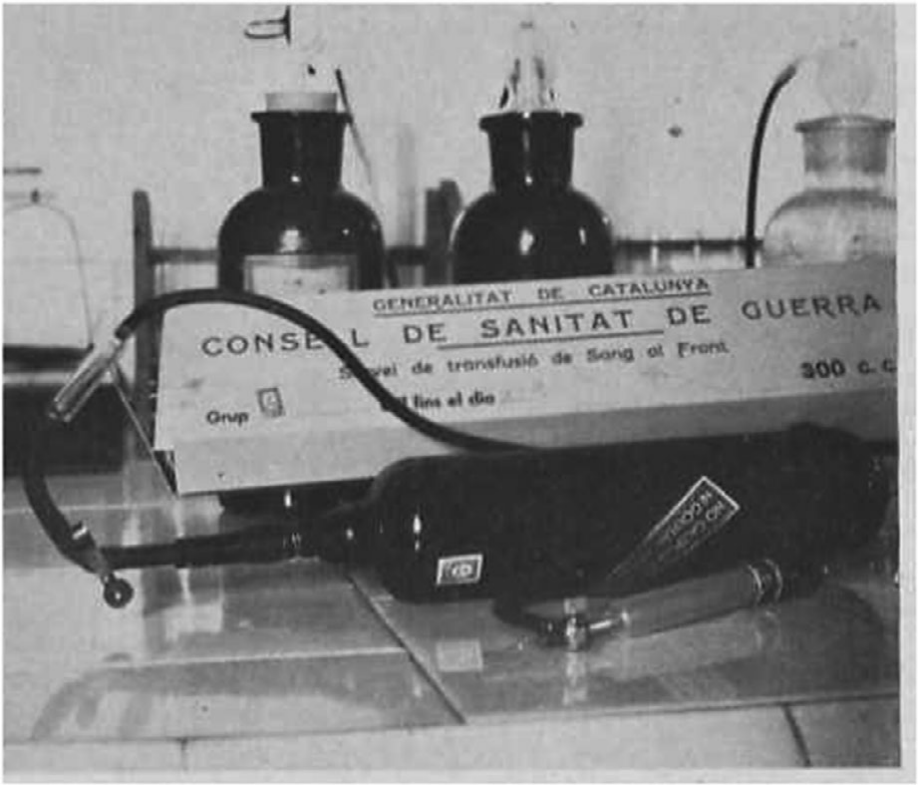
The auto-injectable, 1937. *Source*: Duran Jordà, *op. cit.* (see note 6), Frederic Duran Jordà, ‘El servei de transfusió de sang al front: Organització-Utillatge’ [The Blood TransfusionCentre at the Front: Organization-Tooling], *La Medicina Catalana*, 43-44 (1937), 514.

In the days after the coup d’état of General Francisco Franco in July 1936, Barcelona experienced an episode of extreme violence. Because of the chaos that broke out at the military garrisons and the revolutionary atmosphere that ensued, the health systems had to be improvised by the leftist political parties and partisans.^24^ Duran Jordà was entrusted to organise a blood transfusion centre for attending the wounded in Barcelona at the ‘Hospital de Sangre No. 18’. At first, it did not have enough blood to cover the needs. However, over a few weeks, Duran Jordà was able to take twenty blood samples a day, thanks to the selfless collaboration of the volunteers who responded to public calls broadcast on the radio. In August, the relative pacification of Barcelona and the consolidation of the front in the province of Aragon, 200 km from Barcelona, led Duran Jordà to send blood by car with a refrigeration system.^25^ The innovation of Duran Jordà stemmed from the simplification of the process of extraction from living donors, conservation (following the solution of sodium citrate and refrigeration of Yudin), and the injection with a new device called the auto-injectable (Figure 1), designed by the Laboratori Químic Biològic Pelayo, with a ‘rapide’ patent they had bought from Madrid. The material was given to Duran without economic interest but through the mediation of the Socialist authorities.^26^ In contrast to other devices, it did not require medical specialisation in blood transfusions to use it. Moreover, in October 1936 the Catalan government created a new military health administration (Consell de Sanitat de Guerra) and named Duran’s hospital the Blood Transfusion Service at the Front (Servei de Tranfusió de Sang al Front).^27^

From November 1936, the city of Madrid was endangered by the Francoist troops. In December, the Canadian doctor Norman Bethune set up the Canadian Blood Transfusion Institute (Instituto Canadiense de Transfusión de Sangre), with medical equipment purchased in London and Paris with the funds of the Canadian Committee to Aid the Spanish Democracy (CASD).^28^ It had also a pioneering character in the extraction, preservation, and transport of blood in refrigerated cars. However, because Bethune had bought the medical equipment before knowing Duran Jordà, his technique was less sophisticated: there was no mixing of blood and the preservation bottles were simpler than the auto-injectable.^29^ Both innovators shared the same progressive idea that blood should be donated free of charge in voluntary campaigns through public appeals broadcast on the radio and also delivered to hospitals as a public service. However, the performance of Bethune’s laboratory in Madrid was erratic and Bethune resigned in April 1937, only some months after its foundation. The Institute of Madrid was renamed Instituto Hispano-Canadiense de Transfusión de Sangre and kept operating in the city. From April 1937, the mobile blood transfusion services in wartime Republican Spain were entrusted to Duran Jordà and his method by the Military Health Headquarters (Jefatura de Sanidad Militar). At that time, the first article of his innovative method appeared in a Catalan medical journal, with the first sample of a thousand donors. The Catalan doctor was cautious in his conclusions, emphasising the need to keep investigating.^30^

**Figure 2.**
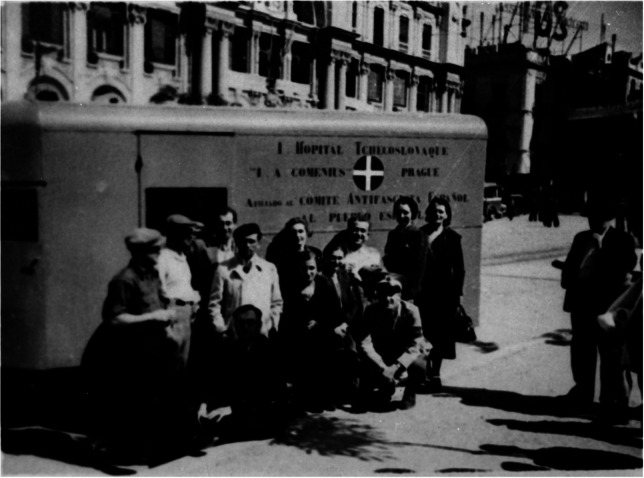
The Komenský Mobile Hospital, 1937. *Source*: *Národní archiv* [National Archives], Prague, collection NAD 1915, Sbírka “Paměti a memoáry – M. Tauchmanová”. Holubec is the first man sitting on the left.

This was the situation when the fourteen-member Czech convoy of the Komenský Hospital crossed the French border and entered Spain (Figure 2).^31^ Karel Holubec was the chief surgeon, followed by the doctors Pavel Bulatý (or Bulatty), Pavel (Avraam) Elik, Josef (Maxim) Laufer, and Vlasta Veselá; the nurses Marie Holubcová (Holubec’s wife), Marie Veselská, Dáda Hůrková-Drozdová, the organizer Otto Schling (Šling), the pharmacist Helena Petránková, the administrator Jan Eisner, the electrotechnician (and later possibly also anaesthetist) Karel Fisher (Fišer), and two drivers: Eugen Doubrava and Karel Vlček. On 9 May, the team joined the International Sanitary Service of the International Brigades and was assigned to the Guadalajara Hospital, near Madrid.^32^ The hospital was located in a garden area of the city and was divided into two areas: one for infectious diseases and the other for the wounded.^33^ The Komenský Hospital also functioned as a diffusion platform, with weekly medical lectures, meetings, and case-study demonstrations.^34^ After the first battle of Guadalajara in March (where Bethune still delivered blood), the military action spread to the eastern mountain ranges of Madrid: Guadarrama, Navacerrada, and El Escorial.^35^ On 15 June 1937, Holubec and other members of the Komenský Hospital were assigned to El Escorial to support the sanitary team of the XI International Brigade, under the orders of another Czech doctor, František Kriegel (1908–1979), and the mobile hospital of Douglas Jolly.^36^

In July, El Escorial became a key hospital during the battle of Brunete, one of the deadliest fights of the Spanish Civil War. The surgical teams worked in exhausting shifts and, despite the short distance from Madrid, the blood used by the surgeons of the International Sanitary Service was provided by Duran Jordà from Barcelona.^37^ Holubec was impressed by the blood transfusion system and the new surgical techniques developed in mobile hospitals by Jolly and Kriegel.^38^ At that time the activity of Duran Jordà reached its peak, with 3,000 registered donors, mostly from Barcelona, coming from different backgrounds: railway labour unions, public servants of the city government of Barcelona, and employees of a department store.^39^

After the battle of Brunete, a new Czechoslovak team arrived in Spain, led by Bedřich Kisch (1894–1968). In August, Holubec met Kisch in Guadalajara and, two weeks later, the Komenský Hospital was relocated to Benicàssim on the Mediterranean coast.^40^ Karel Holubec and his wife Marie, who was pregnant, returned to Czechoslovakia. Shortly after their departure, the doctors of the Komenský Hospital met with Duran Jordà directly in Barcelona.^41^ They likely met after the evacuation of the hospital of Benicàssim that took place in April 1938, under the threat of the Francoists’ advance; all the doctors from that hospital left northwards and spread to other hospitals in the Catalan region.^42^ At that time, the Republicans were losing ground and most of the foreign medical missions were planning to leave the country.

## The transfer

Despite the cosmopolitan environment of the International Brigades and the international exposure of the Spanish Civil War, the innovations of Duran Jordà did not have a remarkable impact on the scientific community. Apart from the Czechs, in July 1937, Duran Jordà received a visit from a Soviet health specialist named (or, rather, codenamed) ‘Colonel Christof’ and, in December, from the British physiologist and activist J. B. S. Haldane, who donated blood for Duran’s laboratory and took one of the devices to the UK.^43^ In February 1938, Duran Jordà was invited to Paris to give a conference at the headquarters of the Centrale Sanitaire Internationale d’Aide à l’Espagne Républicaine (CSIAER), the main organisation which managed the international medical aid to Spain. According to a CSIAER report, by February 1938 Duran Jordà had managed to collect 6,400 litres of blood, from which 1,900 had been delivered to the front, something unprecedented. This was made possible not only by the technical advances of Duran Jordà and his team but also by the frequent campaigns of donation, especially in the biggest cities of Barcelona, Madrid, and Valencia. Furthermore, in its short existence, the lab also improved the auto-injectable, now called ‘Aeron’, which included a two-compartment set.^44^

However, the attempts to export the method of Duran Jordà were confronted by a network of experts who were still wary of experimenting with preserved blood, as was evident at the Second Congress of the International Society of Blood Transfusion held in Paris in October 1937. Duran Jordà’s communications were limited to a leftist audience involved in the Spanish conflict and did not reach mainstream academic circles. In Czechoslovakia, however, the political situation of the country and a massive blood donation campaign organised by associations supporting Republican Spain provoked a huge response from the population and the scientific circles, whose members praised the new Duran Jordà method. This was also possible thanks to the disseminating role of Karel Holubec.

After his return to Czechoslovakia, Holubec shared the knowledge he had acquired as director of the Komenský Hospital with colleagues in seminars and medical journals, where he communicated the basics of the organisation of blood transfusion service in Spain.^45^ In the autumn of 1937, the Society of Friends of Democratic Spain (Společnost přátel demokratického Španělska, SPDŠ – formerly the VPDŠ) carried out extensive campaigns to promote blood donation for Spain through the pages of its monthly magazine *Španělsko* (Spain). The organisation printed 30,000 copies of its first few issues, something quite remarkable by Czechoslovakian standards.^46^ The SPDŠ’s campaign to promote blood donation for the Spanish cause channelled through *Španělsko* was a success. An impressive number of applications arrived at the SPDŠ’s headquarters in a short time: between December 1937 and May 1938, 2,000 people were interested in donating.^47^ Over time, however, the Spanish cause lost ground and blood donation and blood transfusion became more of an internal issue. The editors of *Španělsko* mixed appeals aimed at donating blood to the Spanish and those aimed at donating for the future needs of Czechoslovakia, something apparent in the use of the campaign slogan ‘Blood for Democracy’.^48^ Furthermore, the SPDŠ aimed to ‘hand over the established organisation of blood donors to Czechoslovak institutions’, expressing the hope that it was the duty of the state to ‘take care of the supply of medical services for mass transfusions in cases of emergency, war, epidemics, etcetera.’^49^ In the summer of 1938, 120,000 volunteers had registered by the SPDŠ to donate blood in the ‘Blood for Democracy’ campaign.^50^ Even though signing up for a blood donation did not automatically imply that a person became a donor (we do not know how many of these people actually donated), this was unprecedented and surpassed Duran Jordà’s original campaigns.^51^

In the spring of 1938, during the partial mobilisation of the Czechoslovak army, the Ministry of National Defence appointed some individuals to organise a blood transfusion service. Holubec was chosen to lead, with an improvised character similar to what had happened in Spain, several stations for blood collection and its preservation in the Army.^52^ Although no further sources about these stations, their locations, equipment and procedures are today available, it is not an exaggeration to assume that this was the first *sui generis* transfusion network established in Czechoslovakia. In September 1938, after the general mobilisation had already taken place, ‘a certain amount of preserved blood was created in the Army’ and Holubec’s part in this action is out of doubt.^53^

**Figure 3.**
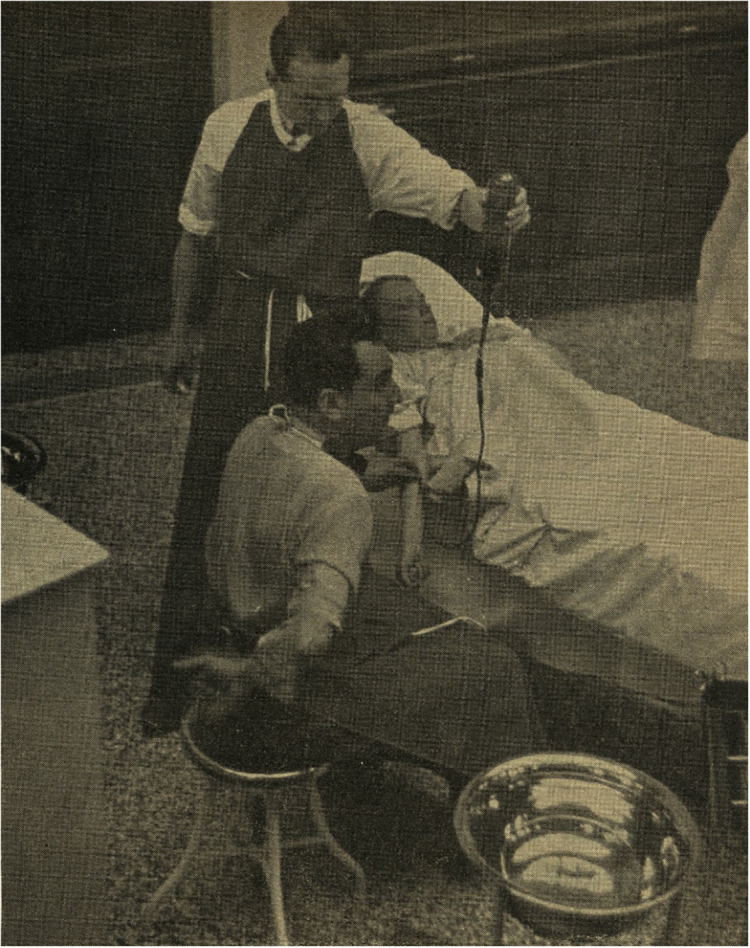
Duran Jordà performing in Czechoslovakia, June 1938. *Source*: Anonymous author, ‘MUDr. Duran v Československu’ [Doctor Duran in Czechoslovakia], *Španělsko*, 2, 4 (1938), 11.

Duran Jordà’s visit to Prague in June 1938 was a major event in Czechoslovakia in the months before the Munich Agreement (Figure 3). His new technique of blood conservation and transfusion and its application in the battlefields of Spain aroused the interest of the local medical community. Duran was invited by the SPDŠ where Holubec was one of the most active members.^54^ Another key player was Captain Emanuel V. Voska (1875–1960), the chairman of the SPDŠ and a prominent social democrat who had left the Austro–Hungarian Empire for the US at the age of nineteen and worked with Tomáš Garrigue Masaryk (later first President of independent Czechoslovakia) during the First World War. In 1919 he returned to Czechoslovakia and, among other activities, collaborated in the foundation of the Czechoslovak Red Cross. In 1936 he was behind the solidarity campaigns in support of the Spanish Republican government.^55^

During his two-week stay, Duran lectured on blood transfusion and demonstrated his method in several cities, under the close attention of hundreds of doctors and representatives of the Ministry of Health and the Czechoslovak Red Cross.^56^ At the first conference in Prague, Spanish Ambassador Jiménez de Asúa introduced Duran to an audience of 600 civilian and military doctors. Duran accompanied his lectures with an instructional film and, then, he ‘demonstrated both his instrumentarium and preserved blood for transfusion, and performed a blood transfusion directly in the lecture hall to a patient, as it is done on the battlefield’.^57^ Discussions were held at the First Czech Surgical Clinic and Bulovka Hospital in Prague (five lectures were given in Prague altogether) and were followed by other lectures in Hradec Králové, Brno, Zlín, and Moravská Ostrava. Duran Jordà performed at least six blood transfusions with blood imported from Spain and all cases bore ‘excellent results’, according to the reports of the time.^58^ On his return to Spain, he shared the following thoughts with a journalist: ‘Today we have told the Czechoslovaks that in eighteen months our blood transfusion service has managed to obtain no less than 4,000 litres of blood from eleven thousand extractions from voluntary donors in our country. This shows both our scientific progress and the magnificent spirit of the citizens of the Spanish Republic’.^59^

Furthermore, Duran Jordà agreed to deliver his patents free of charge, ‘as a gift to Czechoslovak hospitals so that they could increase the strength of our [Czechoslovak] Army and our [Czechoslovak] state’.^60^ The device for filling glass vials with preserved blood (the apparatus) and the method of filling (the procedure) formed a complete ‘system protected by two Catalan patents’, and it was this ‘two-pack’ that was to be made available to the Czechoslovak state.^61^ Duran came with an assistant or collaborator named L. Montagut/Montagud who was the technical director of the Barcelona Institute for Transfusion and also an employee of Cobosa (Comercial Bonin S.A.). Montagut ended up staying in Prague longer in order to assemble the necessary equipment and set up a blood collection apparatus. He taught the medical staff how to fill the bottles with pressure and close them and the equipment was put at the disposal of the State Institute of Health (Státní zdravotní ústav).^62^

However, despite the expectations of the authorities, the medical and military circles, and the considerable publicity which was raised around the planned donation, the handover of the patent fizzled out: ‘Until the day of publication – November 1938 – there has been no written notification about the possibility of free use of the patent, as promised,’ complained one of the interested doctors on the pages of the *Czech Medical Journal.* ^63^ Did Duran as author of the patent, and the firm Cobosa, the trader of the patent, expect a financial profit from the visit to Czechoslovakia even though this was in contradiction to what was originally stated?

It would not be incomprehensible, after all, Cobosa was not a charity organisation, and Duran Jordà could be thinking of a possible source of revenue as the war in Spain continued unfavourably for the Republic. In the early 1940s, Cobosa’s founders, the brothers Joan Lluís and Bonaventura Pujol Font (the latter a surgeon who had served at the front in Catalonia-Aragon), both members of the Catalan Socialist Republican Party Esquerra Republicana de Catalunya (ERC), recognized that they had done their best to sell auto-injectables in Europe but that the war situation made it impossible. They tried to restart the business of auto-injectables in Cuba and suffered economic hardships as exiles.^64^ It is also possible that in the maelstrom of wartime events, both Duran and Cobosa had other concerns to attend to, or could not attend to the petition, and simply dropped the matter. In February 1939, Duran left Spain and migrated to Great Britain, invited by the British Red Cross. He left his wife, Carola Tort, and a two-year-old daughter, Carola Duran, in Barcelona and travelled with the nurse of his lab, Vicenta Villaró, who was pregnant. He was accompanied by Josep Trueta, general surgeon of the General Hospital of Barcelona who became famous for his innovations in the treatment of war fractures.^65^ Shortly after Duran Jordà’s departure, the house of Carola Tort and her daughter was searched by the Francoist police and all suspicious objects (including a doll Duran Jordà had brought to his daughter as a gift from Czechoslovakia) were taken away.^66^

Trueta and Duran Jordà gave detailed accounts of their innovations to the Home Office and the British Army Blood Transfusion Service, without referring to the patent.^67^ In April, Duran Jordà published his main innovations at *The Lancet* and, in 1941, the British Ministry of Information launched a video recognising the innovation of Duran Jordà and showing images of the laboratory of Barcelona.^68^ However, the integration of Duran Jordà and Trueta into the UK was not a bed of roses. The British intelligence often considered the commitment to the Spanish Republic to be indicative of communist affiliation and/or Soviet espionage. Consequently, both doctors encountered significant challenges in their efforts to find a stable position as refugees. They ultimately succeeded: Trueta in Oxford and Duran Jordà in Manchester. The value of their know-how was acknowledged thanks to the imminence of war and the support of some influential doctors who went beyond political suspicion and criticism, like in the case of Trueta, who was supported by doctor Gathorne Robert Girdlestone (1881–1950).^69^ Doctor Janet Vaughan (1899–1993), who lodged Duran Jordà in his first days in London, was paramount in the organization of the Blood Supply Depots in London which, by June 1940, had registered 113,500 volunteer donors through a massive campaign, similar to those conducted in Spain and Czechoslovakia.^70^

## The vicissitudes of Duran’s method in Czechoslovakia

**Figure 4.**
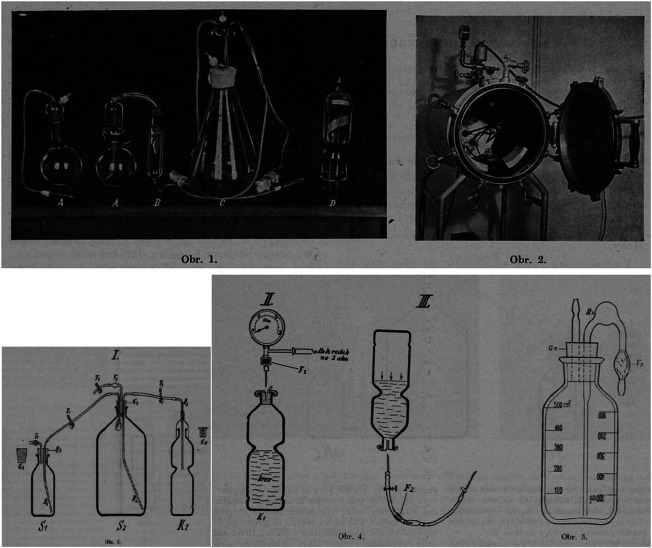
Duran-Drbohlav conservation method, 1938. ‘The principle of the [Duran’s] method is a closed circuit consisting of four vessels (A-D), [figures upper left]. It requires a special apparatus in the form of an autoclave [upper right] into which the pressure of two atmospheres is filled and then the neck of the bottle is sealed. After the bottle is closed, an injection set made of thick-walled hose is fitted over the sealed part, ending in a metal cannula for insertion of the needle … [whereas Drbohlav’s modification consists in] the elimination of thin-walled bottles which are too fragile, and the narrow necks of which cannot be easily cleaned, and their substitution by a thick-walled bottle of the normalised type, which can be easily cleaned as well as in the substitution of negative pressure, which is only available in well-equipped laboratories, by overpressure, which can be easily obtained using a compressor or a bomb … The circuit has been shortened by one container.’ *Source*: *Časopis lékařů českých* [Czech Medical Journal], 77, 44 (1938), 1282-4.

Despite the lack of concretion on the patent transfer, Duran Jordà’s tour in Czechoslovakia had both immediate and long-term effects. Not only was his method widely used, copied, and experimented with, but massive campaigns for blood donation and blood transfusion were organised under his influence.^71^ The opening article of the November 1938 issue of the *Czech Medical Journal* provides a detailed description of how Jaroslav Drbohlav (1893–1946), bacteriologist and the head of the diagnostic department of the State Institute of Health, had modified Duran’s blood transfusion devices ‘in cooperation with Mr Jejkal’ (about whom we have no further information). As a result of this medical–engineering collaboration, they presented a skilful copy of the apparatus and the procedure on the basis of what they had observed in Duran’s demonstrations.^72^ This new method was called the ‘Duran-Drbohlav conservation method’ (Duránova-Drbohlavova konservační metoda, Figure 4) and underwent further modifications in the hospitals of Zlín and Brno.^73^

If Duran’s visit had caused a rising interest amongst surgeons in the use of preserved blood, Karel Holubec was one of the most experienced in this field.^74^ His first experiments with blood transfusion and preservation ‘à la Duran’ took place in Třebíč, a district town in western Moravia, where he had worked since 1936, and where he returned after his Spanish experience. However, after the German occupation of Czechoslovakia, he left the town and moved to Prague, seeking greater anonymity. There, no later than 1942, Holubec found refuge at the Surgery Department of the Vinohrady Hospital under Professor Emerich Polák who was known as the protector and saviour of persecuted patients, often Jews.^75^ Under the auspices and protection of Polák, who himself had experimented with blood transfusions before the war,^76^ Holubec continued with his experiments which followed and adapted Duran’s method, depending on the availability of materials during the war years.^77^

In Prague, Holubec also collaborated with Dr Bohumír Budín^78^ and his resistance group. This group of left-leaning doctors worked within the Czech National Council (Česká národní rada), an illegal resistance body operating in the Protectorate of Bohemia and Moravia. Since 1943, Holubec was instrumental in establishing blood storage and transfusion stations for the Czech resistance, ‘so that the whole of Prague would be secured by this method’.^79^ In the last months of the war he managed to prepare ‘a large amount of blood cans for the wounded and sick in Prague and [the liberated concentration camp of] Theresienstadt.’^80^ An active member of the team which created blood reserves during the Prague Uprising in May 1945 (and which used a blood collection kit provided by the State Institute of Health called ‘Drbohlav’s bottles with vacuum’),^81^ Holubec also took care of the material and financial resources and the training of personnel for the blood transfusion service, mainly relying on his previous experiences from the Spanish battlefields.^82^

In addition to the blood transfusion service, Holubec was actively involved in another crucial activity that should be mentioned in the context of the medical innovations associated with the Second World War, namely the eradication of the typhus epidemic that struck the Terezín/Theresienstadt concentration camp at the end of the war. Although the eradication of the epidemic is a somewhat forgotten story today, it can be argued that it is one of the greatest feats of Czech doctors and medical professionals in Czech modern history. Since April 1945, transports of prisoners from the closed concentration camps elsewhere in Europe began arriving at the Terezín concentration camp by the thousands, suffering from various infectious diseases (most often dysentery and typhoid fever), including typhus fever (louse-borne). The danger of a typhus epidemic was already evident at the end of April but the German camp commanders were more concerned with their own escape, while more evacuation transports arrived in Terezín with groups of prisoners coming from the death marches.^83^

Dr Budín with a group of colleagues had been preparing measures to prevent the spread of contagious diseases as early as the end of 1944. After his death in the Prague Uprising in early May 1945, his work was continued by the Czech epidemiologist Karel Raška and other doctors. Volunteer convoys of health workers began to arrive at Terezín, full of impoverished, dead, and dying prisoners. Among them was Karel Holubec, who had known Raška since school. He may have responded to Raška’s personal appeal, or to the appeals broadcast on the radio which – like the radio appeals for blood in the times of the Spanish Civil War – called on doctors, pharmacists, and nurses to join the aid in Terezín. In mid-May, the epidemic was suppressed by widespread measures and vaccinations. Between the end of April and June 1945, however, around 2,000 people died in Terezín, with typhus fever being among the immediate causes of their deaths.^84^

After the Second World War Duran Jordà’s contributions were not forgotten in Czechoslovakia. In 1948, the *Czech Medical Journal* published a study about blood transfusions by the aforementioned epidemiologist Raška who referred to Duran Jordà, ‘a name known to all of us’ and his role in the development of blood transfusions in Great Britain.^85^

At the beginning of 1948, the Czechoslovak Ministry of Health, instructed by the Ministry of National Defence, established the so-called Transfusion Commission, and by a government resolution of 7 December 1948, the health administration of the now communist state built a network of transfusion stations.^86^ The first stations began operating in the spring of 1949 in Prague and Brno. In the summer, other stations appeared, such as in the relatively small district town of Třebíč.^87^ In the Transfusion Commission which administered these stations, Holubec was well positioned for being ‘our leading expert with international experience’, not only from Spain but also from the Soviet Union, where he had also made a study trip in 1938.^88^ Furthermore, in the spring of 1950, the Transfusion Commission came under the direct leadership of the Deputy Minister of Health, a military physician and former boss of Holubec in Spain, Dr František Kriegel. After Spain, Kriegel served at China’s Red Cross Medical Relief Corps and in the medical army of the Allied Forces in China, India, and Burma.^89^ Upon his return to Czechoslovakia, he entered politics and became an organiser of the People’s Militias (Lidové milice) and an active player in the process of the communist takeover.^90^

Holubec’s career skyrocketed, too. Soon after the liberation of Czechoslovakia in 1945, he officially joined the Communist Party (having sympathised with it since much earlier), left Prague in early 1946, and returned to Třebíč where he became head of the surgery department.^91^ In Třebíč he fully developed his experience as a war surgeon from Spain specialising in open fractures, burns, and blood transfusions. He became an associate professor and lectured on war surgery at Masaryk University in Brno.^92^ His wife Marie, who had practiced as an operating theatre nurse in Spain and had since long been active in the Czechoslovak Red Cross, decided to devote herself exclusively to the household and family.^93^

Marie’s decision to withdraw from the Red Cross activities may of course be read in the personal context of a woman who just gave birth to their third child, whose husband was often absent from home, and whose domestic helper was taken away by the local authorities after the communist coup in February 1948, but also in the context of the new refugee policy of the communist state: although at first glance the Czechoslovak Red Cross represented a guarantee of continuity with pre-war practice in the care of refugees, in fact after 1948 it fell fully under the control of the Communist Party and functioned as a transmission lever between the International Section of the Central Committee of the Communist Party of Czechoslovakia and the state security apparatus, which kept an eye on the foreigners (at the moment mostly Greeks, but also Italians, Yugoslavs, and Spaniards). In the eyes of local communist authorities, Marie may not have appeared to be the most suitable, i.e. politically reliable, person to be employed in such an organisation.^94^

Holubec renewed his contacts with Czech and Slovak comrades from Spain when the Society of Friends of Democratic Spain resumed its activities. At the same time, members of the Spanish Republican exile began to arrive in Czechoslovakia enjoying extraordinary support, as did – for the time being – the Czechoslovak International Brigaders, who were hailed as heroes for having taken up the fight against fascism before it was fully unleashed in Central Europe.

In 1947 and 1948, as a scholarship holder of the Ministry of Health, Holubec went on several months of trips to France (autumn 1947) and England (autumn 1948), the aim of these trips being, *inter alia*, to study medical innovations in the field of blood transfusion.^95^ It cannot be ruled out that he met Duran while in England. By then, Duran Jordà was working at the Ancoats Hospital in Manchester and was in touch with other Republican exiles, who visited him and praised his work, giving him the Prat de la Riba award (1947). However, in a 1949 letter to a friend, he defined himself as the ‘outcast of Manchester’ and he laughed at himself as he had once gained fame as a cold-blood doctor (because of the preserved blood) and now he was in need of heating his blood, as he was not getting used to the cold-weathered Manchester.^96^ He passed away in 1957 at the age of 51.

At the turn of 1948–49, in connection with another planned study trip to the UK, Holubec was contacted for the first time by Czechoslovak intelligence and was offered free access to the West in exchange for an agreement to cooperate. Although we know, in view of later developments, that Holubec was not suitable for cooperation with the secret services and that he was unable to carry out the given tasks (mainly because of his scientific absent-mindedness and impracticality, and possibly, too, his personal reluctance), he did not turn the agent down. At the same time, however, he himself became an object of interest for the State Security (Státní bezpečnost).^97^

Around the same time that the State Security opened a file on him, Holubec was engaged in two projects of his own, in the spirit of humanist-oriented socialism and socialist internationalism: the first was to make a promotional documentary about blood transfusion and its preservation. The second was the promotion of Gypsy/Romani culture and the public empowerment of Czechoslovak Gypsies/Roma.

As far as the documentary film was concerned, the intended format was not dissimilar to the one Duran Jordà had presented in Prague in 1938. The largest medical institutions in Czechoslovakia still referred to the original video that Duran Jordà had shown in the summer of 1938 as a solid source of knowledge.^98^ How the idea for this project was born and developed, is revealed by a unique source, which is Holubec’s communication with Vítězslav Nezval, a prominent writer, poet, leading figure of Czech surrealism, and pre-war communist who became very active politically after the Second World War.^99^ The men were friends. In February 1949, Holubec turned to Nezval for help. He attached a film script, ‘as I drafted it between evening and midnight work’, to promote blood donation and support the idea of establishing a network of transfusion stations.^100^ He asked Nezval to review his script and help promote it, as the poet had connections to the highest political circles. At the same time, the doctor admitted that ‘the whole Blood Transfusion Commission is working unsatisfactorily slowly’.^101^ Later that year, a fifteen-minute film, entitled ‘Dar nejcennější’ [A Most Precious Gift] was produced for promotional and educational purposes, matching the format that Holubec envisioned.^102^ Whether Nezval’s ‘cultural patronage’ was behind the making of this film, as Holubec kept suggesting, or not, the film, as a medium whose popularity grew steeply after the war, certainly played a significant role in promoting the idea of donation and blood transfusion.

The doctor’s relationship with the Czechoslovak Roma is another aspect of Holubec’s personality demonstrating – just as much as the documentary project – his struggle for greater social justice after the war. The Roma who lived during the war in the territory of the Protectorate of Bohemia and Moravia and the Sudetenland, i.e. the Czech part of the former Czechoslovakia, overwhelmingly perished in the Holocaust. After the war, Slovak Roma began to migrate en masse to the Czech lands, especially to border areas and large cities, in search of job opportunities. Although formally equal to the rest of the population, their integration was difficult and the communist regime struggled to respond effectively to these challenges, resulting in various policy proposals ranging from repressive measures to support for Roma self-government.^103^ Holubec sought to shift public discourse towards enlightenment and openness, promoting the development of Roma culture and their active participation in public life. From 1950 until well into the 1960s, he studied Roma culture, customs, and language and although he was an autodidact, he gradually became a recognised expert.^104^ Holubec visited Roma families in Brno and Třebíč, advocating for their integration and proposing to the Communist Party ways to involve the Roma in the construction of socialism. He criticized racial discrimination and believed that Roma should be allowed to develop their culture and language, proposing the use of Romani for educational and political materials and even translating and publishing Roma folk poetry.^105^

The penetration of Soviet ideology into Czechoslovak science and medicine, which, according to several scholars, met with some resistance in 1948–49, gained momentum from the turn of the year 1950–51, and Czechoslovak science found itself under the growing isolation from the other side of the Iron Curtain. Only a few individuals or small groups were able to cross borders and there were explicit efforts to expel ‘deeply rooted Western influences’ from Czech medical science.^106^ Sovietisation was spread using a variety of methods: the introduction of planning into scientific research and the attempts at centralisation; propaganda campaigns in the scientific press; the influence of Soviet advisors; the transfer of organisational models in higher education and the Czechoslovak Academy of Sciences; pressure for translations of Russian literature; and the necessary ‘shielding’, i.e. the very carefully monitored number of citations from Soviet literature. In the Czechoslovak medical press, Soviet production displaced the studies and monographs by Anglo–Saxon authors that had dominated until then.^107^

By 1951, the Transfusion Commission was criticised (and criticised itself) for being led by ‘reactionaries’, Western-oriented cosmopolitans, Trotskyists, or Jews: ‘It turned out that our transfusion service has been built on erroneous foundations according to Western experience and needs to be rebuilt according to the Soviet model’.^108^ As a result, the activities of the Commission were paralysed and many non-conforming or independent experts were laid off and left the country (most of them illegally and irreversibly). Meanwhile, in small and medium-sized Czechoslovak hospitals transfusions were done with a pre-Duran method with a syringe without citrate directly from the donor’s vein into the patient’s vein, or the donor’s blood was allowed to drain into an open container with citrate, where it was stirred with a whisk and then injected into the recipient with a syringe.^109^ The blood transfusion service continued to be voluntary but with some rewards in terms of food and other product allowances. A 1951 report informed that ‘often there are defective canned meats, chocolate is wormy, tea is missing and complaints from affected donors are not given a hearing’.^110^ The food package had to be ‘covered’ by a reference to the Soviet Union and its ‘perfectly established system of voluntary blood donation’.^111^

**Figure 5.**
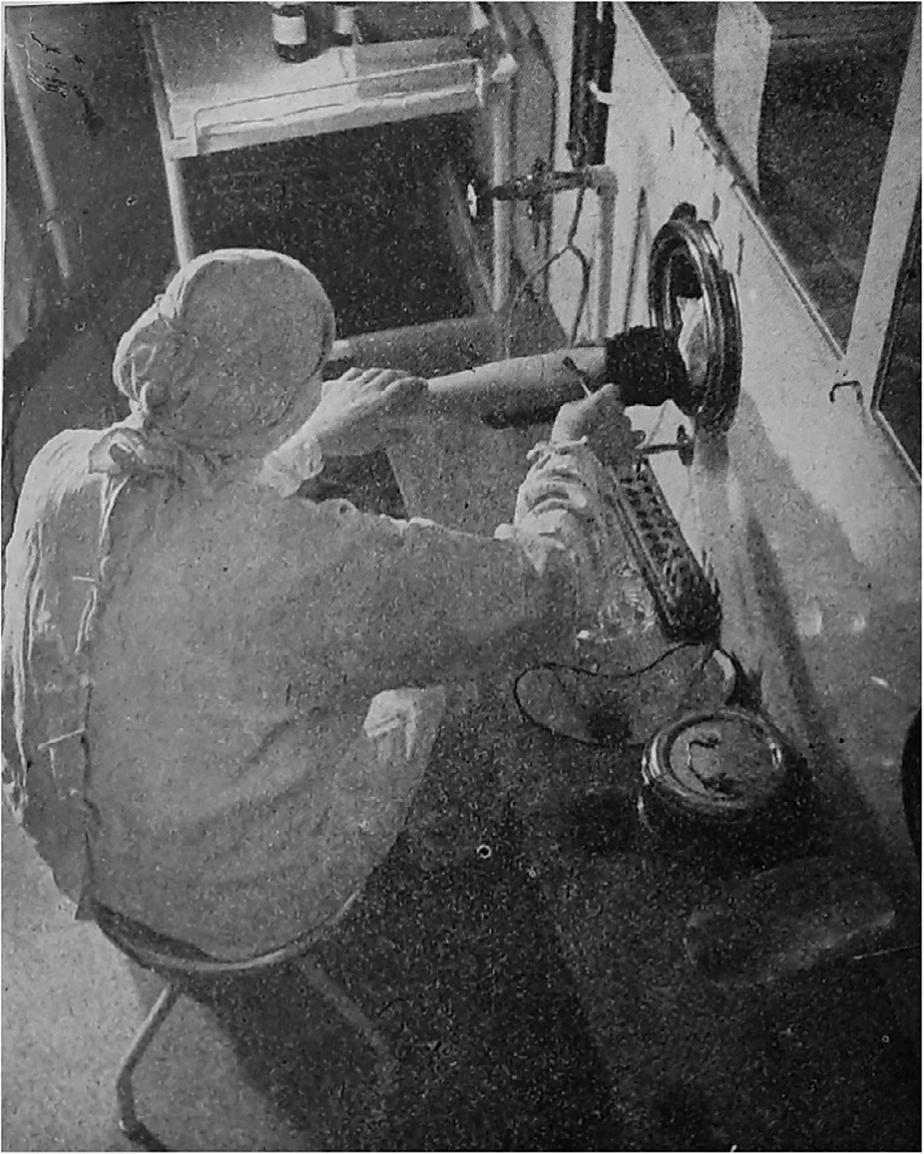
Aseptic box of the transfusion station in Czechoslovakia, mid-1950s. *Source*: Eduard Dobrý and Jaroslav Fiala, *Dárcovství krve* [Blood Donation] (Prague: Ministerstvo zdravotnictví, 1957), 62.

On the other hand, the network of transfusion stations began to expand, as did the number of voluntary donors, and blood transfusion gradually established itself as a functioning part of the health system of communist Czechoslovakia. The dense network, as conceived by Soviet expertise in view of the risks of a possible nuclear war, guaranteed a good blood supply to all medical facilities. The examination of donors was improved, and the safety of transfusions was increased (again largely due to the Russian emphasis on aseptic practice, Figure 5).^112^

In the climate of political purges and scientific isolation of Czechoslovakia from Western Europe, Duran Jordà was remembered as a distinctive and enduring figure, as a Republican emigrant, who stood somewhat outside the divide of the Cold War. It is within this context, too, that we must read the relative longevity of Duran’s practices in Czechoslovak medicine. His findings were referred to by Czechoslovak experts in the treatment of human blood well into the mid-1960s.^113^ By that time, Holubec stepped out of his involvement in the institutions of blood transfusions and ceased publishing and lecturing in this field.^114^ In the late 1950s and the early 1960s, he worked in Congo, Algeria, and Ethiopia on various Czechoslovak development aid missions. Despite the isolation between Eastern and Western Europe, Czechoslovakia, and other Communist countries, he engaged in diverse international activities with other territories from both the Socialist world and with countries of the so-called Third World. Although we do not know whether Duran’s blood transfusion method had a role in Holubec’s work in Africa, he used Trueta’s method of treating splinter fractures, with which he had become familiar in Spain, ‘with fair results, even in combat situations’^115^ in Algeria, where he was sent a member of a Czechoslovak medical expedition to help the rebel Algerians in the last months of the struggle for a free Algerian state,^116^ or the 1960 Congo at the time of Patrice Lumumba.^117^ Having retired from his position as chief medical officer in 1967, Holubec died of cancer in 1977. His wife Marie Holubcová (1913–2004) lived to see the fall of the communist regime and, in 1996, became an honorary citizen of Spain.^118^

## Conclusions

The beginnings of blood transfusion and blood conservation in Czechoslovakia were strongly influenced by the expertise of the Catalan doctor F. Duran Jordà, whose method was diffused by Duran himself and a group of experts led by Karel Holubec, a volunteer doctor who had experienced how this method was applied in wartime Spain. During the Second World War, this method was further transformed and adapted in some Czechoslovak hospitals. References to Duran’s method persisted in post-war and communist Czechoslovakia well into the 1950s, despite the influx of expertise from the Soviet Union. However, as much as the Czechoslovak blood transfusion and its actors tried to keep up with world developments in the 1950s and 1960s, the isolation from Western Europe and the imposed Sovietisation caused a growing technological backwardness and the method of Duran Jordà was overlaid and covered by other influences. Today, his contribution to Czech and Slovak medical histories, while unquestionable, has been largely forgotten.

Duran Jordà and Holubec were both heavily influenced by the political milieu in which they worked: the Spanish Civil War and occupied Czechoslovakia. Their methodology implied massive campaigns of solidarity which were broadcast in the media and received strong popular support, bringing blood to their laboratories for free and being allocated by public and military administrations who were facing a situation of war. Their ‘socialist’ understanding of medicine and blood transfusion was determined by the military needs of two countries that were under the pressure of a total war. Furthermore, they both developed an internationalist approach, which enabled them to transport blood from both countries and link both blood transfusion campaigns in Spain and Czechoslovakia. This was unprecedented and pioneered in the international solidarity campaigns that would develop after the Second World War.

Duran Jordà’s transfer to Czechoslovakia has not been registered or recognised as a relevant step in the history of blood transfusions partly due to its semi-peripheral character. The main tools of transmission, besides the direct performance, were publications in Catalan, Spanish, French, and Czech which appeared in journals and bulletins of leftist organisations that did not reach a wider scientific audience, even though – as this article has shown – they could be massively followed by citizens. Meanwhile, the mainstream international medical community did not recognise the value of Duran Jordà’s innovation, at least in the key years of 1937–39, which makes the transfer from Spain to Czechoslovakia even more relevant in historical terms. Thus, if the revision of Duran Jordà’s method transfer can fall in the ‘novelty-mongering’ trend that is common in the historians of science, technology, and medicine, it is also a ‘history of content and context together’^119^, a history from below and a case study that conflates the innovation and its dissemination with the history of wartime Spain and Czechoslovakia. The Spanish Civil War, the International Brigades, and the invasion of Czechoslovakia by Nazi Germany were thus not only the historical context of medical innovation but an integral part of it.

This article also shows a process of invisibilisation and paths not taken. The Second World War and the first years of the Cold War implied a reconfiguration of the international scientific networks and contacts, affecting the relationship of Duran Jordà and Holubec. After Duran Jordà had migrated to the UK, although his identity as a Spanish Republican refugee was not without problems, his method was absorbed by the Western international medical community. In contrast, Holubec and other Czechoslovak doctors kept working and adapting Duran Jordà’s method according to their needs, but their channels of communication, especially during the German occupation, were narrowed. When the Sovietisation campaigns became pressing in the 1950s, these channels were further censored and cut off from their original source, although new waves of communication were opened with other territories, which remain outside the possibilities of this article. As a consequence, the transfer of Duran Jordà’s method to Czechoslovakia and its relevance in the international history of blood transfusions seemed to enter into a dead-end road.

## Footnotes

1 On medical innovations during the Spanish Civil War, see Various Authors, *Los médicos y la medicina en la guerra civil española* [The Doctors and Medicine in the Spanish Civil War]. (Madrid: Beecham, 1986); Felip Cid, *La contribució científica catalana a la medicina i cirurgia de guerra (1936-1939)* [The Catalan Scientific Contribution to Medicine and War Surgery (1936-1939)]. (Barcelona: Fundació Uriach 1838, 1996); Nicholas Coni , Medicine and Warfare. Spain, 1936-1939 (Oxfordshire: Routledge, 2008).

2

Susan Lederer , Flesh and Blood: Organ Transplantation and Blood Transfusion in Twentieth Century America (Oxford: Oxford University Press, 2008), 89.

3

Paul Preston , ‘Two Doctors and One Cause: Len Crome and Regginald Saxton in the International Brigades’, International Journal of Iberian Studies, 19, 1 (2006), 5–25 ; Carles Brasó Broggi , Los médicos errantes: De las Brigadas Internacionales y la revolución china a la guerra fría [Doctors from Nowhere: From the International Brigades and the Chinese Revolution to the Cold War] (Barcelona: Crítica, 2022); See also Francisco Guerra, *La medicina en el exilio republicano* [The Medicine in the Republican Exile] (Alcalá de Henares: Universidad de Alcalá, 2003).

4

Natalie Zemon Davis , ‘Decentering History: Local Stories and Cultural Crossings in a Global World’, History and Theory, 50, 2 (2011), 188–202 ; See also Roger Cooter, Mark Harrison, Steve Sturdy (eds.), *War, Medicine and Modernity* (Stroud, Sutton Publishing, 1999).

5 On biographical aspects of Duran Jordà, see Carola Duran i Tort , ‘Frederic Duran Jordà i el servei de transfusió de sang’ [Frederic Duran Jordà and the Blood Transfusion Centre], Gimbernat, 20 (1993), 83–90 ; Joan Grífols i Espés, *Frederic Duran i Jordà: Un mètode, una época* [Frederic Duran Jordà: One Method, One Epoch] (Barcelona: Hemo-Institut Grífols, 1997); José Ramón Navarro Carballo, *Frederic Duran Jordà: Un hito de la historia de la transfusión sanguínea* [Frederic Duran Jordà: A Milestone in the History of Blood Transfusion]. (Madrid: Ministerio de Defensa, 2005).

6 Frederic Duran Jordà, ‘El servei de transfusió de sang al front: Organització-Utillatge’ [The Blood Transfusion Centre at the Front: Organization-Tooling], *La Medicina Catalana*, 43-4 (1937), 512-16.

7

Frederic Duran Jordà , ‘El servicio de transfusión de sangre de Barcelona. Técnicas y utillaje’ [The Blood Transfusion Centre of Barcelona. Techniques-Tooling], Revista de Sanidad de Guerra, 1, 8 (1937), 307–21; Carles Hervás and Manuel Cahisa i Mur, ‘Notas históricas sobre el hospital de sangre número 18 de Barcelona (1936-1939)’ [Historical Notes on the Blood Hospital No. 18 in Barcelona (1936-1939)], *Gimbernat*, 27 (1997), 173-84.

8

Moisès Broggi , ‘Sobre Frederic Duran Jordà’ [On Frederic Duran Jordà], Gimbernat, 27 (1997), 185–91; Josep Maria Massons, ‘L’obra de Frederic Duran Jordà viscuda per mi’ [The Work of Frederic Duran Jordà as I remember], *Revista de la Reial Acadèmia de Medicina de Catalunya*, 21, 2 (2006), 52-5; see the special issue at the *Revista de la Reial Acadèmia de Medicina de Catalunya*, 21, 2 (2006). See also Miguel Lozano and Joan Cid , ‘Frederic Duran-Jorda: A Transfusion Medicine Pioneer’, Transfusion Medicine Reviews, 21, 1 (2007), 75–81 17256247 10.1016/j.tmrv.2006.08.004; Linda Palfreeman, *Spain bleeds: The Development of Battlefield Blood Transfusion During the Civil War* (Brighton, Sussex Academic Press, 2015).

9 Karel Holubec, ‘Od Guadalajary do Benicasimu’ [From Guadalajara to Benicassim], in *Rok práce a zkušeností lazaretu Jan Amos Komenský v demokratickém Španělsku* [A Year of Work and Experiences of the Komenský Hospital in Democratic Spain] (Prague: Společnost přátel demokratického Španělska, 1938), 16.

10

Carles Brasó Broggi , ‘The International Sanitary Service (ISS) and the Innovations in Mobile Hospitals and Auto-Chirs during the Spanish Civil War, 1936–1939’, in Paloma Fernández Pérez (ed.), Business History of Hospitals in the 20th Century (New York: Springer, 2024), 71–82.

11 See John Pickstone (ed.), *Medical Innovations in Historical Perspective* (Basingstoke: Palgrave Macmillan, 1992); Jennifer Aniston, *Innovations in Health and Medicine. Diffusion and Resistance in Twentieth Century* (London: Routledge, 2002); Ilana Löwy, Olga Amsterdamska, John Pickstone, Patrice Pinell (eds.), *Medicine and Change: Historical and Sociological Studies of Medical Innovation* (Paris: INSERM, 1993). We take the concept of semi-peripheries from Immanuel Wallerstein, *The Modern World-System I: Capitalist Agriculture and the Origins of the European World-Economy in the Sixteenth Century* (Cambridge: Academic Press Inc., 1974), where Spain and Eastern Europe remain central to his argument.

12 Duran Jordà, *op. cit.* (see notes 6 and 7). In 1937 he published a propaganda book in English titled *The Service of Blood Transfusion at the Front: Organization, Apparatus* (Barcelona: Comissariat de Propaganda de la Generalitat de Catalunya). In December 1937, there was a special issue of the Spanish *Revista de Sanidad de Guerra* (Vol. 8) dedicated to Duran’s method; fragments of it were also translated into other languages at internationalist journals like the *Bulletin Centrale Sanitaire Internationale d’Aide à l’Espagne Républicaine* or *AMI – Periódico de la Ayuda Médica Extranjera.* His method was published in English in 1939: F. Duran Jorda, ‘The Barcelona Blood-Transfusion Service’, *The Lancet*, 233 (1939), 773-5.

13 See Linda Palfreeman and Peter H. Pinkertone, ‘Transfusion in the Spanish Civil War: Supply and Demand, the Role of the “Blood Transfusion Officer” and British Planning for the Outbreak of the Second World War’, *Transfusion and Apheresis Science*, 58, 6 (2019); Linda Palfreeman and Peter H. Pinkertone, ‘El servicio de transfusión del ejército republicano durante la Guerra Civil Española y su influencia en Gran Bretaña en la Segunda Guerra Mundial’ [The Blood Transfusion Centre of the Republican Army during the Spanish Civil War and Its Influence in Great Britain in the Second World War], *Asclepio* 74, 1 (2022), 1-11.

14

A. D. Farr , ‘Blood Group Serology: The First Four Decades (1900-1939)’, Medical History, 23 (1979), 215–26.381816 10.1017/s0025727300051383PMC1082436

15 For Czechoslovakia, see Miloš Netoušek, *Krevní převod* [Blood Transfer] (Prague: Nakladatelství Spolku českých lékařů 1945), 140-1; Josef Nedvěd *et al.*, ‘Léta 1920–1950: Začátky české krevní transfuse’ [Years 1920–1950: The Beginnings of Czech Blood Transfusion], *Transfuze a hematologie dnes*, 12, 2 (2006), 109.

16

Sergei Sergeevich Yudin , ‘Transfusion of Stored Cadaver Blood’, The Lancet, 230, 5946 (1937), 362-6.

17

Moisès Broggi , Memòries d’un cirurgià (1908-1945) [Memoirs of a Surgeon] (Barcelona: Edicions 62, 2001), 112–3.

18 Palfreeman, *op. cit* (see note 8), 21.

19 Cid, *op. cit.* (see note 1), 388-9.

20 Broggi, *op. cit.* (see note 8), 186.

21 Josep L. Ausin Hervella, ‘Frederic Duran Jordà i els projectes d’assegurança social a la malaltia’ [Frederic Duran Jordà and the Projects of Social Insurance for the Sick], *Revista de la Reial Acadèmia de Medicina de Catalunya*, 21 (2006), 56-61.

22 Josep Trueta, ‘Frederic Duran Jordà. Un innovador entusiasta’, undated, newspaper clipping, Arxiu Nacional de Catalunya (*National Archive of Catalonia*, hereafter ANC), ANC1-901-T-45, Dossier sobre l’activitat en el camp de les transfusions durant la guerra civil espanyola del doctor Frederic Duran i Jordà.

23

Antonia Rodrigo García , Doctor Trueta: Héroe anónimo de dos guerras [Doctor Trueta: Anonymous Hero of Two Wars] (Barcelona: Base, 2009), 50.

24 Duran Jordà, *op. cit.*, (see note 7), 307.

25 Anonymous, ‘Las transfusiones de sangre podrán hacerse en las trinchera’ [The Blood Transfusions Will Be Possible in the Trenches], *La Libertad*, October 25, 1936, 4.

26 Autobiografia i missatge pòstum de Lluís Marcó i Dachs [Autobiography and Posthumous Message of Lluís Marcó i Dachs], Oral Testimony, ANC1-563-N-558. See also Carolina Helbig Cerezo and Eduardo Mª Targarona i Soler, ‘La transfusión sanguínea durante la Guerra Civil española’, 1978, in ANC1-901-T-46.

27 Hervàs Puyal & Cahisa Mur, *op. cit.* (see note 7).

28 Norman Bethune (Servicio Canadiense de Transfusión de Sangre) to Benjamin Spence, 17 December 1936, reproduced in Roderick Stewart, *Norman Bethune* (Ontario: Fitzhenry & Whiteside, 1977), 50-5.

29

Roderick Stewart and Sharon Stewart , Phoenix: The Life of Norman Bethune (Montreal: Mc-Gill-Queen’s University Press, 2011), 163–5 and 413.

30 Duran Jordà, *op. cit.* (see note 6), 516.

31 Jaroslav Bouček, ‘Los médicos checoslovacos en la guerra de España’ [The Czechoslovak Doctors in the Spanish War], *Ibero-Americana Pragensia*, 37 (2003), 161-9; Carles Brasó Broggi, *op. cit.* (see note 3), 22.

32 Anonymous, ‘Medical Aid from Czechoslovakia to Republican Spain’, New York University Taminent Library & Wagner Labor Archives, *Abraham Lincoln Brigade Archives* (hereafter *ALBA*), Fredericka Martin Papers, Box 15, Folder 18: Benicassim undated, 1937-1938, 1970-1977; for the list of the medics’ names see also Holubec, *op. cit.* (see note 9), 5, 16.

33 ‘Testimonio de Steffi Wenzel a Gusti Jirku’ [Testimony of Steffi Wenzel to Gusti Jirku], Rossijskij gosudarstvennyj arhiv social’no-politicheskoj istorii [Russian State Archive of Socio-Political History], (hereafter *RGASPI*), fond Interbrigady respublikanskoj armii Ispanii [Collection Interbrigades of the Spanish Republican Army], 545/2/188, 250-3.

34 Bedřich Kisch, ‘Lazaret Komenský v Benicasimu’ [The Komenský Hospital in Benicassim], *Španělsko*, 1, 10 (1937), 8.

35 Stewart and Stewart, *op. cit.* (see note 29), 197-9.

36 Base de las Brigadas Internacionales, 11a Brigada, Estado Mayor, June 15, 1937. Archivo General Militar de Ávila [Ávila’s General Military Archive], AGMAC, C.1092, 1/79, 90.

37 On the blood provision to El Escorial, see Broggi, *op. cit.* (see note 17), 218; and José M. Massons, *Historia de la sanidad militar Española. Tomo II* [History of Spanish Military Health. Volume II] (Barcelona: Ediciones Pomares-Corredor, 1994), 355.

38 Holubec, *op. cit.* (see note 9), 16; See also Emanuel Viktor Voska, ‘Dvojí účel’ [Double Purpose], *Španělsko*, 2, 2 (1938), 10.

39 Anonymous, ‘Tres mil donadores de sangre para los heridos de guerra’ [Three Thousand Blood Donors for the Wounded of War], *La Vanguardia*, 15 July, 1937, 3.

40 Anonymous, *op. cit.* (see note 25); See also *Vojenský ústřední archiv–Vojenský historický archiv* [Central Military Archive] (hereafter *VÚA/VHA)*, Prague, fond [collection] (hereafter f.) 255, personal file of Bedřich Kisch no. 52277.

41 ‘Dáváme si zkoušet krev i pro Čechoslováky: Přednáška F. Buriana do barcelonského rozhlasu’ [We’re Having Our Blood Tested for Czechoslovaks as well: Lecture by F. Burian for Barcelona Radio], *Španělsko*, 2, 4 (July 1938), 10; See also K. Holubec, ‘Lékařské zkušenosti ze španělské války’ [Medical Experiences from the Spanish War], *Přítomnost*, *nezávislý týdeník*, 15, 13 (1938), 199.

42 Guillermo Casañ, ‘Evacuación del hospital de las Brigadas Internacionales de Benicàssim a Cataluña’, in R. Monlleó (ed.), *Castelló al segle XX* [Castelló in the Twentieth Century] (Castelló, Universitat Jaume I, 2006), 499-546; See also Brasó Broggi, *op. cit.* (see note 3), 83-8.

43 Anonymous, *op. cit.*, (see note 25), 3; Anonymous, ‘Un sabio inglés da sangre para los combatientes’ [An English wiseman gives blood for the fighters], *La Vanguardia*, December 24, 1937, 5.

44 Bibliothèque Nationale de France, ‘La transfusion de sang conservé’ [Transfusion of Stored Blood], *Centrale Sanitaire Internationale d’Aide a l’Espagne Républicaine. Bulletin d’Information* (1938), 20-3.

45 Karel Holubec, ‘K léčení střelných poranění humeru’ [On Treatment of Gunshot Wounds of the Humerus], *Rozhledy v chirurgii*, 16, 1-2 (1937), 20-4; *idem*, ‘Úkoly krevní transfuse v poli’ [Blood Transfusion Tasks in the Battlefield], *Rozhledy v chirurgii*, 16, 8 (1938), 319-24. In the pages of this surgical journal, Holubec also reviewed many scientific studies, often those published in *Revista de Sanidad de Guerra* and in Russian journals. He copied photographs so that readers could see what Spanish cars for blood transport, refrigerators, etc. looked like (see *ibid.)*

46 See *Národní archiv* [National Archives] (hereafter *NA*), Prague, f. 225 Ministerstvo vnitra I – prezidium [Ministry of Interior – Presidium], karton [box] (hereafter box) 1269, sign. 225-1269-4/90, ‘Výroční zpráva Výboru pro demokratické Španělsko za rok 1937’ [Annual Report of the Committee for Democratic Spain for 1937], 3; See also [Anonymous] ‘P. T. předplatitelům!’ [To Distinguished Subscribers!], *Španělsko*, 1, 5 (1937), 15. *Španělsko* has so far been the only extant source providing partial insight into the activities of the Czechoslovak Committee to Aid the Democratic Spain (VPDŠ, later SPDŠ). The magazine is preserved in the National Museum Library in Prague and the authors would like to thank Mr Alberto Ortiz for making available the digital form of selected issues.

47 See the brief note next to the article [Anonymous] ‘Dárci krve republice a demokracii’ [Blood Donors to the Republic and Democracy], *Španělsko*, 2, 2 (1938), 9.

48 K. Holubec, ‘Krevní transfuse ve španělské armádě’ [Blood Transfusions in the Spanish Army], *Španělsko*, 2, 2 (1938), 10; Voska, *op. cit.* (see note 38).

49 Voska, *op. cit.* (see note 38).

50 Anonymous, ‘Věrnost za věrnost’ [Loyalty for Loyalty], *Španělsko*, 2, 4 (1938), 1.

51 As for the real impact of the campaign, there seems to be no way to reconfirm it in terms of responsiveness, as there are no official statistics. Various vectors must be mentioned. First, the editors of *Španělsko* may have wanted to exaggerate the success of the campaign. Second, a high number could have been achieved due to the considerable circulation of *Španělsko*, the frequency with which the appeals were published, and the fact that the SPDŠ also used other channels of communication. Last but not least, there is the logic of how health campaigns work in moments of crisis like in the dramatic times of late summer 1938 in Czechoslovakia. In this light, the figure of 120,000 interested donors may not look so fantastic.

52
*Moravský zemský archiv* [Moravian Provincial Archives] (hereafter *MZA*), Brno, fond Krajský národní výbor Jihlava [Regional National Committee Jihlava], box 1024, inv. č. [inventory number] (hereafter inv. no.) 2186, correspondence between Holubec and the Provincial Office in Brno in the matter of granting the post of chief physician in Třebíč or in another hospital, attached CV, 13 November 1945; See also Arnold Jirásek, ‘Poznámky dnešního chirurga k převodu krevnímu’ [Today’s Surgeon’s Notes on Blood Transfer], *Časopis lékařů českých*, 87, 48 (1948), 1242. According to Jirásek, Holubec organised five centres for the collection and preservation of blood in the Army.

53 Eduard Dobrý, *Životodárná tekutina* [Life-Giving Liquid] (Prague: Ústřední výbor ČČK, 1967), 15.

54 Grífols, *op. cit.*, (see note 5) 158; Jaroslav Drbohlav, ‘Příspěvek k problému konservace krve’ [A Contribution to the Problem of Blood Conservation], *Časopis lékařů českých*, 77, 44 (1938), 1281.

55 On E. V. Voska see Dagmar Hájková, *Emanuel Voska: Špionážní legenda první světové války* [Emanuel Voska: The Spy Legend of the First World War] (Prague: Academia, 2014).

56 Anonymous, ‘Lékařské společnosti – přednášky’ [Medical Societies – Lectures], *Časopis lékařů českých*, 77, 23 (1938), 727.

57 Anonymous, ‘Zpráva o konání XXX. vědecké schůze lékařů v. v. nemocnice hl. m. Prahy na Bulovce’ [Report on the Thirtieth Scientific Meeting of the Doctors of the General Public Hospital of the Capital City of Prague at Bulovka], *Časopis lékařů českých*, 77, 26 (1938), 809. Today, only a fragment of the film is available. It was reproduced in 1941 by the UK Ministry of Information. The video is available at https://wellcomecollection.org/works/hb7wfbuz [accessed 22 June 2024], the part of Duran Jordà starting at minute 08:28.

58 Anonymous, ‘Zpráva o přednášce “Konservování krve a krevní transfuse” MUDr. F. Durána, šéfa služby krevní transfuse v Barceloně, konané v posluchárně I. české chirurgické kliniky dne 17. června 1938’ [Report on the Lecture “Preservation of Blood and Blood Transfusion” by Dr F. Duran, Head of the Blood Transfusion Service in Barcelona, Held in the Auditorium of the First Czech Surgical Clinic on 17 June 1938], *Vojenské zdravotnické listy*, 14, 4 (1938), 318-21; Anonymous, ‘Zpráva o konání XXX. vědecké schůze lékařů v. v. nemocnice hl. m. Prahy na Bulovce [Report on the Thirtieth Scientific Meeting of the Doctors of the General Public Hospital of the Capital City of Prague at Bulovka], *Časopis lékařů českých*, 77, 26 (1938), 809; Anonymous, ‘MUDr. Durán v Československu’ [Doctor Duran in Czechoslovakia], *Španělsko*, 2, 4 (1938), 11; Drbohlav, *op. cit.* (see note 54), 1281.

59 Anonymous, ‘La obra sanitaria de la República Española en el Extranjero’ [The Health Work of the Spanish Republic Abroad], undated, newspaper clipping, ANC1-901-T-45.

60 Anonymous, ‘MUDr. Duran v Československu’ [Doctor Duran in Czechoslovakia]), *Španělsko*, 2, 4 (1938), 12; Grífols, *op. cit.* (see note 5), 157.

61 Drbohlav, *op. cit.* (see note 54), 1282-6; E. V. Voska, ‘Po roce’ [One Year Later], *Španělsko*, 2, 4 (1938), 8-9.

62 Both spellings (Montagut/Montagud) appear in the press at the time. We do not know the full first name.

63 Drbohlav, *op. cit.* (see note 54), 1282.

64 Letter from Bonaventura Pujol Font to Andreu Abelló, 2 January, 1942, ANC1-408-T-1291.

65 Rodrigo, *op. cit.* (see note 23), 22-8.

66 Testimony of Carola Duran. We would like to thank Carola Duran for her sympathy and willingness to share her thoughts as a young child.

67 The National Archives (Kew Gardens, London), ‘Air-raid precautions department, Home Office: Spanish Civil War air-raid casualties, 1939’, FD, 1/5372.

68 Blood Transfusion (1941), see note 57.

69 Rodrigo, *op. cit.* (see note 23), 26, 115.

70 Palfreeman and Pinkertone, *op. cit.* (see note 13), 6.

71 Nedvěd et. al*., op. cit.* (see note 15), 108-10; Jirásek, *op. cit.* (see note 52), 1241.

72 Drbohlav, *op. cit.*, (see note 54), 1281-6. The main modification consisted of two things: 1) replacing the thin-walled and thus fragile and breakable bottle with thick-walled glass, and 2) substituting negative pressure with overpressure.

73 Netoušek, *op. cit.* (see note 15), 141-3.

74
*Ibid.*, 142. Another example would be Bohuslav Voženílek, who in the mid-1930s was interested in Yudin’s technique of transfusing preserved blood from corpses. See Bohuslav Voženílek, ‘Transfuse konservované krve’ [Transfusion of Preserved Blood], *Časopis lékařů českých*, 76, 45 (1937), 1825-31.

75 Ludmila Hlaváčková and Hana Mášová, ‘Z pamětí prof. MUDr. Emericha Poláka’ [From the Memoirs of Professor Emerich Polák], *Práce z dějin Akademie věd*, 2, 2 (2010), 131-48, here 131-2; Bedřich Placák, *Paměti lékaře* [A Doctor’s Memoirs] (Prague: Torst, 1997), 193-4.

76 Petr Svobodný, ‘Die Hämatologie an den Prager Medizinischen Fakultäten vom Ausgang des 19. bis zur Mitte des 20. Jahrhunderts’ [Haematology at the Prague Medical Faculties from the End of the Nineteenth to the Middle of the Twentieth Century], in Jiří Pešek and Falk Wiesemann (eds), *Blutt: Perspektiven in Medizin, Geschichte und Gesellschaft* [Blood: Perspectives in Medicine, History and Society] (Essen: Klartext, 2011), 228; See also Emerich Polák, *Paměti* [Memoirs] [typewritten manuscript, mid-1970s, kept in the Institute of History of Medicine, First Faculty of Medicine of Charles University, no. N 1037], 137-8.

77 Netoušek, *op. cit.* (see note 15), 143-4. Netoušek emphasises that ‘Holubec was particularly careful about aseptic processing – the preparation for blood collection having to be the same as for surgery –, about the free draining of blood and storage in a refrigerator’.

78 Bohumír Budín (1907–45) was a member of the team which, at the end of the Second World War, developed the first Czech antibiotic (Mykoin BF510). Martin Franc, *Úderná skupina? Výprava českých lékařů a přírodovědců do SSSR v roce 1950 ve světle dopisů Ivana Málka* [A Strike Group? The Expedition of Czech Doctors and Natural Scientists to the USSR in 1950 in the Light of Ivan Málek’s Letters]. (Prague: MÚA, 2009), 23.

79
*VÚA/VHA*, f. 255, personal file of Karel Holubec no. 27969, folder ‘Podklady k žádosti o vydání Osvědčení podle zák. č. 255/1946 Sb.’ [Documents Supporting the Application for the Issue of the Certificate according to Act No. 255/1946 Coll.].

80 Dobrý, *op. cit.* (see note 53), 15.

81 M. Stolzová-Sutorisová, J. Liška, and K. Klinderová, ‘Určování krevních skupin en masse a příprava konserv lidské krve v revolučních dnech’ [Determination of Blood Groups en masse and Creating Human Blood Reserves in Revolutionary Days], *Časopis lékařů českých*, 85, 47 (1946), 1638-41, here 1640.

82
*VÚA/VHA*, f. 255, personal file of Karel Holubec no. 27969, folder Documents Supporting the Application for the Issue of the Certificate according to Act No. 255/1946 Coll., Letter to Jos. Havlíček, Employee of the Secretariat of the Central Committee of the Communist Party of Czechoslovakia, 3. 3. 1966; Confirmation by MUDr. Konopík, 7. 1. 1966; Confirmation by MUDr. Špaček, 28. 3. 1966; Holubec’s own statement in his biographical material and other documents.

83

Miroslava Benešová , ‘Likvidace tyfové epidemie a osvobození Terezína’ [Eradication of the Typhus Epidemic and Liberation of Terezín]. In Václav Novák (ed.), Malá pevnost Terezín [Small Fortress Terezín] (Prague: Naše vojsko, 1988), 232–48.

84 Benešová, *op. cit.* (see note 83), 240; see also Tomáš Staněk, *Poválečné ‘excesy’ v českých zemích v roce 1945 a jejich vyšetřování* [Post-war ‘Excesses’ in the Czech Lands in 1945 and Their Investigation] (Prague: Ústav pro soudobé dějiny AV ČR, 2005), 157-9.

85

Karel Raška , ‘Organisace transfusní služby ve Velké Británii’ [The Organisation of the Transfusion Service in the UK], Časopis lékařů českých, 87, 48 (1948), 1253.

86 The official name was the Commission for the Organisation of the Transfusion Service in the Czechoslovak Republic (Komise pro organizaci transfusní služby v ČSR). *NA*, f. 315/1 Úřad předsednictva vlády–běžná spisovna 1945-1959 [Office of the Presidency of the Government–common files, 1945-1959] (hereafter ÚPV-B 1945-1959), inv. no. 5370, sign. 1452/61, box 1220, folder Zřízení nové transfusní služby [Establishment of a New Transfusion Service], ‘Informace pro pana přednostu odboru, věc: Národní transfusní služba’ [Information for the Head of the Department, Matter: National Transfusion Service], 21. 4. 1949. By order of the Ministry of Health of 27 November 1948, sixteen Transfusion Stations (TS) were established at regional hospitals in Prague, České Budějovice, Hradec Králové, Ústí n. L., Plzeň, Havlíčkův Brod, Brno, Olomouc, Třebíč, Ostrava, Zlín, Bratislava, Košice, Nitra, Turč. Sv. Martin, and Báňská Bystrica. At the beginning of 1950, eleven stations were operating with a total of 210 employees. In Slovakia, the TS began operating later than in the Czech lands. See *ibid.*, Minutes of the Seventh Meeting of the Commission for the Organisation of the Transfusion Service in Czechoslovakia Held on 27 and 28 January 1950, dated 9. 3. 1950; *ibid.*, Letter of Ministry of Health to the Office of the Presidency of the Government, in the hands of Dr Kučera, 2.

87
*MZA*, f. Krajský národní výbor Jihlava [Regional National Committee Jihlava], box 1062, inv. no. 2362, folders ‘Investiční a projektové úkoly pro okres Třebíč’ [Investment and Project Tasks for the Trebic District] and ‘Výstavba Státní okresní nemocnice v Třebíči 1946-1953’ [Construction of the State District Hospital in Trebic 1946-1953]; *ibid.*, box 1039, inv. no. 2241, folder ‘Zestátnění léčebných a ošetřovatelských ústavů v jihlavském kraji’ [Nationalisation of Medical and Nursing Institutions in the Jihlava Region] and ‘Úprava služebních a platových poměrů 1949-1950’ [Adjustment of Service and Salary Conditions 1949-1950]. Holubec was head of the Surgery Department in Třebíč from 1946.

88
*NA*, f. 315/1 ÚPV-B 1945-1959, inv. no. 5370, sign. 1452/61, box 1220, folder ‘Establishment of a New Transfusion Service’, Information for the Head of the Department, Matter: National Transfusion Service, 21. 4. 1949, 2; *ibid.*, ‘Zpráva o výrobě sušené krevní plasmy’ [Report on the Production of Dried Blood Plasma], 30. 1. 1950, 2.

89 Brasó Broggi, *op. cit.* (see note 3), 135-228.

90
*NA*, f. 315/1 ÚPV-B 1945-1959, inv. no. 5370, sign. 1452/61, box 1220, Letter of Ministry of Health, Vice-Deputy Dr Kriegel to Office of the Presidency of the Government, ‘Zpráva o stavu transfusní služby na území ČSR’ [Report on the State of the Transfusion Service in the Czechoslovakia], 12. 7. 1950, 4. See also Martin Groman , ‘Continuity – My Main Impulse for Participating in the Movement’: The Role of Political and Social Ideals in the Life Story of František Kriegel, Soudobé dějiny/Czech Journal of Contemporary History, 30, 3 (2023), 785–817 ; Hana Bortlová, ‘El trienio cubano de un asesor checo – La Cuba revolucionaria en los diarios del médico y político František Kriegel’ [The Cuban Triennium of a Czech Advisor - Revolutionary Cuba in the Diaries of the Physician and Politician František Kriegel], in Josef Opatrný (ed.), *Las relaciones entre Europa Oriental y América Latina 1945–1989* (Prague: Karolinum, 2015), 147–56.

91
*MZA*, f. Krajský národní výbor Jihlava, box 1024, inv. no. 2186, Zemský NV v Brně Ministerstvu zdravotnictví, věc: V. v. nemocnice v Třebíči, Určení MUDra K. Holubce za primáře chirurgického oddělení [Provincial National Committee in Brno to the Ministry of Health, Matter: Hospital in Třebíč, Designation of Dr K. Holubec as Head of the Surgical Department], 6. 2. 1946.

92
*Seznam přednášek, které se budou konati na Masarykově universitě v Brně* [List of Lectures to be Held at Masaryk University in Brno], 1947-48 Winter (Brno: Masarykova universita, 1947), 18; *ibid.*, 1948-49 Winter (Brno: Masarykova universita, 1948), 21.

93
*MZA*, f. Krajský národní výbor Jihlava, box 1105, inv. no. 2621, folder ‘Evidence zdravotnických pracovníků, přihlášky, okres Třebíč 1950-57’ [Register of Medical Workers, Applications, Třebíč District 1950-57], MUDr. Holubec – zaměstnání manželky [Dr Holubec Wife’s Employment], February 1950.

94 According to the testimony of a State Security officer, Marie Holubcová refused to continue her service in the Czechoslovak Red Cross because she ‘supposedly had no time’ and, according to the same writer, she was not only ‘politically completely inactive’ but also behaved and acted ‘in a purely bourgeois manner’. *Archiv bezpečnostních složek* [Security Services Archive], Prague (hereafter *ABS*), fond Hlavní správa rozvědky SNB-svazky [Main Intelligence Directorate of the National Security Corps-files], reg. no. 41987 I. S (code name ‘Havel’), Doplněk k memorandu [Addendum to memorandum] MUDr. Doc. Holubec-Havel, 19. 9. 1955, 2. For insights into the Czechoslovak state’s refugee policy after 1948 see Doubravka Olšáková, V krajině za zrcadlem: Političtí emigranti v poúnorovém Československu a případ Aymonin [Behind the Mirror: Political Emigrants in post-1948 Czechoslovakia and the Aymonin Case], *Soudobé dějiny*/*Czech Journal of Contemporary History*, 14, 4 (2007), 719-43, here 724-5.

95
*ABS*, Prague, f. Zpravodajská správa Generálního štábu Československé lidové armády-svazky [Intelligence Administration of the General Staff of the Czechoslovak People’s Army-files] (hereafter ZS/GŠ), archivní číslo [archival number] (hereafter arch. no.) OS-16211 ZSGŠ Holubec Karel, MUDr. docent – informátor: Úřední záznam [Dr Holubec Karel, Associate Professor, Informant: Official Record], 10. 9. 1948; K. Holubec , ‘Služba krevního převodu ve Francii’, Časopis lékařů českých, 87, 48 (1948), 1250–2.

96 Letter from Duran Jordà to Joan Triadú, 26 November 1949, ANC1-667-T-815, ‘Frederic Duran Jordà’.

97
*ABS*, f. ZS/GŠ, arch. no. OS-16211 ZSGŠ Holubec Karel, úřední záznam [Official Record], 10. 9. 1948; *ABS*, f. Hlavní správa rozvědky SNB-svazky [Main Intelligence Directorate of the National Security Corps-files], reg. no. 41987 I. S (code name ‘Havel’), Doplněk k memorandu [Addendum to memorandum] MUDr. Doc. Holubec -Havel, 19. 9. 1955. The reason to open a file on Holubec was presumably because of his contact with the aforementioned E.V. Voska. In 1946 Holubec asked Voska to arrange a surgical course in London for him. Voska, an agent of the American intelligence service and after the war, the arch-enemy of the people’s democratic establishment, was arrested in 1950 and sentenced to ten years’ imprisonment in a trial in 1954. On the tragic fates of many Czechoslovak and other Eastern European interbrigadists in the Stalinist trials of the early 1950s, see, e.g. Ádám Anderle , ‘El Calvario de los interbrigadistras húngaros’, Acta Hispanica 18 (2013), 63–71 ; Jaroslav Bouček, ‘Českolovenští interbrigadisté jako zdroj politických elit po roce 1945’, in Ivana Koutská and František Svátek (eds), *Politické elity v Československu 1918-1948* (Prague: Ústav pro soudové dějiny AV ČR, 1994), 147-80.

98 A. Jirásek, *op. cit.* (see note 52), 1242.

99 Vítězslav Nezval (1900–58) was a peer of Holubec and grew up in the Třebíč region where, in the post-war period, he acted as a kind of cultural patron of the Communist Party. It is possible that Nezval and Holubec first met in Třebíč before the war. In the late 1930s, several Czech avant-garde artists with whom Nezval was friends (Toyen, Štýrský, Teige, the Surrealist group ‘Devětsil’, etc.) were involved in supporting Republican Spain. For these people, Holubec could have represented a ‘symbol’ of Czechoslovak medical aid to Spain.

100
*Literární archiv Památníku národního písemnictví* [Literary Archive of the National Literature Memorial] (hereafter *LA PNP*), Prague, f. Nezval Vítězslav, 1186, folder ‘Korespondence vlastní, přijatá’ [Personal Correspondence, Received], Holubec Karel - Nezval Vítězslav, 1954-1957, Letter of K. Holubec to V. Nezval, 7. 2. 1949. However, the text of the script itself is not available in the collection.

101
*Ibid.*

102 Written and directed by Jan Svoboda, length fifteen minutes (Prague: Krátký film and Czechoslovak Red Cross, 1949). To reveal further details about the possible collaboration of Holubec, or Nezval for that matter, in the production of the film would require more research. The authors would like to thank Marie Barešová from the National Film Archives in Prague for her help in searching for the film. See also *LA PNP*, f. Nezval Vítězslav, 1186, folder ‘Korespondence vlastní, přijatá’, Holubec Karel - Nezval Vítězslav, 1954-1957, Letter of K. Holubec to V. Nezval dated 7. 2. 1949, 2.

103 Nina Pavelčíková, ‘Historie Romů v českých zemích v letech 1945-1989’ [History of the Roma in the Czech Lands 1945-1989], In Martin Kaleja and Jan Knejpl (ed.), *Mluvme o Romech* [Let’s talk about the Roma] (Ostrava: Ostravská univerzita 2009), 41-3.

104 Michal Schuster, Svaz Cikánů-Romů: První romská organizace v českých zemích [Union of Gypsies and Roma: The First Romani Organization in the Czech Lands]. In Kaleja and Knejpl, *op. cit.* (see note 103), 53.

105 Ivo Balík, *Z kapsy bílého pláště, aneb, Půlstoletí s medicínou: padesát nahodilých zastavení z mé lékařské praxe s jedním dodatkem* [From the pocket of the white coat, or, half a century with medicine: fifty random stops from my medical practice with one addition] (Pelhřimov: Nová tiskárna Pelhřimov, 2010), 47. See also *NA*, Prague, A ÚV KSČ [Czechoslovak Commnist Party Central Committee Archives], f. 05/3 (Ideological Department), volume 36, archival unit 294, Návrh Karla Holubce ÚV KSČ [Proposal by Karel Holubec to the Central Committee of the Communist Party of Czechoslovakia], 5. 3. 1953, quoted in Matěj Spurný, *Nejsou jako my: Česká společnost a menšiny v pohraničí 1945-1960* [They Are Not Like Us: Czech Society and Minorities in the Borderland 1945-1960] (Prague: Antikomplex, 2011), 275. Karel Holubec [under the pseudonym Jan Sobota], *Cikán zpívá jinak* [Gypsies Sing Differently] (Prague: Československý spisovatel, 1954).

106 Franc, *op. cit.* (see note 78), 53; About sovietisation see Jan Hálek (ed.), *Ve znamení „bdělosti a ostražitosti“. Zahraniční styky a emigrace pracovníků ČSAV v dobových dokumentech (1953–1971)* [Under the Sign of ‘Vigilance and Alertness’. Foreign Relations and Emigration of Workers of the Czechoslovak Academy of Sciences in Contemporary Documents, 1953–1971] (Prague: MÚA, 2011); Martin Franc and Věra Dvořáčková *et al.*, *Dějiny Československé akademie věd I 1952-1962* [History of the Czechoslovak Academy of Sciences I 1952-1962] (Prague: Academia, 2020); Milena Josefovičová, *Z Československé akademie věd do exilu. S vědci o vědě* [From the Czechoslovak Academy of Sciences to Exile. With Scientists about Science] (Prague: MÚA, 2011). Soňa Štrbáňová and Antonín Kostlán (eds.), *Sto českých vědců v exilu: encyklopedie významných vědců z řad pracovníků Československé akademie věd v emigraci* [One Hundred Czech Scientists in Exile: an Encyclopaedia of Prominent Scientists from the Czechoslovak Academy of Sciences in Exile] (Prague: Academia, 2011). Another interesting source on sovietisation in science in general and medicine in particular are memoirs, see Otto Wichterle , Vzpomínky [Memories] (Prague: Evropský kulturní klub, 1992); Otakar Poupa, *Syndrom kolibříka: neveselé kapitoly o vědě a moci, aneb šedesát let zkušeností* [Hummingbird Syndrome: Some Unhappy Chapters about Science and Power, or Sixty Years of Experience] (Prague: Galén, 2000); Josef Charvát and Miroslava Sedloňová (ed.), Můj labyrint světa: vzpomínky, zápisky z deníků [My Labyrinth of the World: Memories, Diary entries] (Prague: Galén, 2005).

107 Franc, *op. cit.* (see note 78), 45-7.

108
*NA*, f. 315/2, Úřad předsednictva vlády–tajná spisovna 1945-1959 [Office of the Presidency of the Government-secret files, 1945-1959] (hereafter ÚPV-T 1945-1959), inv. no. 2302, sign. 285/2, box 583, ‘Informace pro pana předsedu vlády. Věc: Výroba sušené krevní plasmy a zpráva o celostátní konferenci NTS’ [Information for the Prime Minister. Matter: Production of Dried Blood Plasma and Report on the Conference of the National Transfusion Service], 15. 10. 1951, signed by A. Kučera, 1-2, 4.

109 Nedvěd et. al*., op. cit.* (see note 15), 109.

110
*NA*, f. 315/2, ÚPV-T 1945-1959, inv. no. 2302, sign. 285/2, box 583, Informace pro pana předsedu vlády. Věc: Výroba sušené krevní plasmy a zpráva o celostátní konferenci NTS, 15. 10. 1951, signed by A. Kučera, 2.

111
*Ibid.* Such ‘shielding’ (i.e. any mention in Soviet literature, procedures in experiments, etc.) in efforts to promote a particular view or procedure was another common, if paradoxical, phenomenon accompanying the process of Sovietisation in Czechoslovakia. See Franc, *op. cit.* (see note 78), 11-12.

112 While in 1956 there were 158 medical posts within transfusion service in the country, the number grew to 175 by the end of 1958. The number of blood transfusions almost quadrupled between 1950 and 1955. See *Československé* z*dravotnictví ve statistických údajích 1948-1958* [Medical Yearbook 1948-1958] (Prague: Státní zdravotnické nakladatelství, 1958), *passim*; Eduard Dobrý and Jaroslav Fiala, *Dárcovství krve* [Blood Donation] (Prague: Ministerstvo zdravotnictví, 1957), 5.

113

J. Mysliveček , M. Vrkočová and H. Jeničková , ‘Vztah mezi vlivem heparinu a hyaluronidasy při srážení krve’ [Relationship between the Effect of Heparin and Hyaluronidase in Blood Clotting], Časopis lékařů českých, 92, 5 (1953), 126–3113042866 ; J. Vacl , K. Bílá and M. Princ , ‘Hematogenní oxidační terapie’ [Haematogenic Oxidative Therapy], Časopis lékařů českých, 105, 7 (1966), 183–4, 1875906105 .

114 Holubec’s main interest in this field was blood transfusions in connection with the treatment of stomach ulcers. He published on this topic until the second half of the 1950s. His last related article is most likely K. Holubec , ‘Indikace pomalého krevního převodu’ [Indications of Slow Blood Transfer], Vojenské zdravotnické listy, 26, 7 (1957), 312–6. See also K. Holubec , Krvácení z gastroduodenálního vředu a jeho chirurgické léčení [Gastroduodenal Ulcer Bleeding and Its Surgical Treatment] (Prague: Státní zdravotní nakladatelství, 1955).

115 Karel Holubec, *Blíž k obratníku: Chirurg v tropech* [A Surgeon in the Tropics] (Prague: Avicenum, 1976), 319.

116
*ABS*, fond Hlavní správa rozvědky SNB-svazky [Main Intelligence Directorate of the National Security Corps-files], reg. no. 41987 I. S (code name ‘Havel’).

117 Czechoslovak experts sent to the newly decolonized African states became an integral part of the Czechoslovak development project in the Global South, especially in the 1960s. Their task was to properly represent the achievements of Czechoslovak socialism and disseminate them. They were usually engineers, geologists, agronomists, or brewers but doctors and nurses were also very common. From the 1970s onwards, however, the rhetoric of socialist solidarity and the desire to export socialist modernity began to give way to economic pragmatism. See Jan Koura and Mikuláš Pešta , ‘Československo a studená válka v Africe’ [Czechoslovakia and Cold War in Africa], Paměť a dějiny, 14, 3 (2020), 3–13 . It will be possible to clarify the circumstances of Holubec’s stay in Africa once the archival materials of the then Ministry of Health are fully available.

118 Various documents and photographs from Holubec family archive, courtesy of Jiří Holubec; V. Šerý , ‘Zemřel doc. MUDr. K. Holubec’, *Časopis lékařů českých* , 116, 5 (1977), 159–60; Josef Kroupa , ‘Vzpomínka na doc. MUDr. Karla Holubce’, *Úrazová chirurgie* , 5, 1 (1997), 32–3.

119

David Edgerton , ‘Innovation, Technology, or History: What Is the Historiography of Technology About?’ Technology and Culture, 51, 3 (2010), 680–94.

